# Combined utilization of metabolic inhibitors to prevent synergistic multi-species biofilm formation

**DOI:** 10.1186/s13568-022-01363-4

**Published:** 2022-03-04

**Authors:** Dingrong Kang, Wenzheng Liu, Fatemeh Bajoul Kakahi, Frank Delvigne

**Affiliations:** 1 TERRA Research and Teaching Centre, Microbial Processes and Interactions (MiPI), Gembloux Agro-Bio Tech, University of Liège, 5030 Gembloux, Belgium; 2 School of Food and Pharmaceutical engineering, Nanjing Normal University, 210000 Nanjing, China

**Keywords:** Multi-species, Biofilm formation, Biofouling, Wastewater, Metabolic inhibitor

## Abstract

Biofilm is ubiquitous in industrial water systems, causing biofouling and leading to heat transfer efficiency decreases. In particular, multi-species living in biofilms could boost biomass production and enhance treatment resistance. In this study, a total of 37 bacterial strains were isolated from a cooling tower biofilm where acetic acid and propionic acid were detected as the main carbon sources. These isolates mainly belonged to *Proteobacteria* and *Firmicutes*, which occupied more than 80% of the total strains according to the 16S rRNA gene amplicon sequencing. Four species (*Acinetobacter* sp. CTS3, *Corynebacterium* sp. CTS5, *Providencia* sp. CTS12, and *Pseudomonas* sp. CTS17) were observed co-existing in the synthetic medium. Quantitative comparison of biofilm biomass from mono- and multi-species showed a synergistic effect towards biofilm formation among these four species. Three metabolic inhibitors (sulfathiazole, 3-bromopyruvic acid, and 3-nitropropionic acid) were employed to prevent biofilm formation based on their inhibitory effect on corresponding metabolic pathways. All of them displayed evident inhibition profiles to biofilm formation. Notably, combining these three inhibitors possessed a remarkable ability to block the multi-species biofilm development with lower concentrations, suggesting an enhanced effect appeared in simultaneous use. This study demonstrates that combined utilization of metabolic inhibitors is an alternative strategy to prevent multi-species biofilm formation.

**Supplementary Information:**

The online version contains supplementary material available at 10.1186/s13568-022-01363-4.

## Key Points

- 37 bacterial strains were isolated and identified from a cooling tower biofilm.
- Synergistic effect of biofilm formation was observed among four species.
- Three metabolic inhibitors showed effective inhibition against biofilm formation.
- Targeting cellular metabolism is an effective way to inhibit biofilm formation.

**Supplementary Information:**

The online version contains supplementary material available at 10.1186/s13568-022-01363-4.

## Introduction

Diverse microorganisms co-exist and form biofilms with emerging properties, such as enhanced nutrient acquisition, antibiotic tolerance, and internal cooperation in response to environmental stress (Elias and Banin 2012; Flemming et al. 2016; Karygianni et al. 2020). However, microbial biofilms can cause serious biofouling with concomitant efficiency reductions in industrial water systems, like cooling towers (Bott 1998). Water sources for cooling systems are commonly derived from groundwater or surface water (AM Peer and Sanders 2016). Recently, there is an increasing interest in using municipal or plant wastewater to replace freshwater consumption, because of environmentally beneficial and sustainable development (Li et al. 2011). Nevertheless, wastewater has abundant nutrient substances, such as volatile fatty acids (acetate, propionate, and butyrate). They are the essential intermediates in microbial fermentation processes, resulting in plentiful microbial growth and biofilm formation (Zhou et al. 2018). Therefore, seeking an effective strategy to reduce biofilm formation is crucial to prevent biofouling.

Microbial communities in cooling towers are highly dynamic, while they generally share taxa associated with biofilm formation (Tsao et al. 2019). A common core microbiome was identified from biofilms of four cooling towers filled with different water sources (Di Gregorio et al. 2017). In particular, *Pseudomonas* was acknowledged to be within the pioneer colonizers, following more species involved in forming multi-species biofilms (Doğruöz et al. 2009). Interspecific interactions including cross-feeding and metabolic exchange among mixed biofilms enhance biomass production, facilitate the microbes to survive in harsh environments, and increase resistance to external stress (Tan et al. 2017). Hence, targeting key species related to biofilm forming rather than free-living cells has been found effective to block biofouling.

Several approaches have been developed to limit biofilm formation in cooling water systems. The most frequent countermeasure is by adding biocides to target essential components, such as the cellular walls, membranes, structural proteins, and RNA/DNA (Di Pippo et al. 2018). However, microbes residing in biofilms display much higher tolerance to these biocides compared to free-living cells (Fux et al. 2005; Harrison et al. 2007). On the other hand, material modifications have shown their efficacy to prevent the initial biofilm attachment in the cooling pipes by coating with nanoparticles (Ogawa et al. 2016). Moreover, imidazolium and piperidinium-based ionic liquids were found to inhibit cell adhesion to different materials, reducing biofilm formation (Anandkumar et al. 2020; Reddy et al. 2017). Other methods such as using the quorum quenching bacteria (Jo et al. 2016), ultrasonic treatments (Rodríguez-Calvo et al. 2020), and electromagnetic processes (Xiao et al. 2020) have been explored to obstruct biofilm development or eliminate the formed biofilms. Nevertheless, few of them are implemented with great success to field antifouling applications due to the limitations in terms of strain specificity, stability, and long-term efficacy (Flemming 2020).

Metabolic inhibitors are able to target specific microorganism pathways, acting on diverse gene expression regulative cascades and impacting metabolic functions (Mohiuddin et al. 2020; Saiardi et al. 2018). Different inhibitors were used against specific phenotypical traits like biofilm formation (Cho et al. 2020; Lohse et al. 2020). Regarding the latter, various metabolic pathways were reported to associate with biofilm development in recent years (Armbruster and Parsek 2018; Rabin et al. 2015). For instance, a previous study has illustrated that second messenger cyclic di-GMP (c-di-GMP) guides the bacterial switching behavior from a planktonic state to a biofilm formation lifestyle (Valentini and Filloux 2016). In this study, we investigated the efficacy of metabolic inhibitors on the multi-species biofilm formation from industrial water systems. Cultivable bacteria were isolated and identified, and synergistic biofilm communities were constructed from representative strains. Subsequently, three metabolic inhibitors: sulfathiazole (ST), 3-bromopyruvic acid (3BP), and 3-nitropropionic acid (3-NP) were employed to prevent the biofilm formation of multi-species. The present work is expected to provide an alternative strategy to overcome biofouling based on targeting cellular metabolism related to biofilm formation.

## Materials and methods

### Biofilm sample and synthetic medium

A biofilm sample was collected from a cooling tower located in a French industry plant that produces sugar and alcohol. The cooling tower was fed by wastewater with a pH range from 7.0 to 9.0 (28 °C). The main carbon sources found in wastewater were volatile fatty acids, including propionic acid (1.50 g/L), acetic acid (1.00 g/L), lactic acid (0.016 g/L), and formic acid (0.013 g/L). A synthetic medium was used to mimic the cooling tower environmental conditions. Synthetic medium was based on M9 minimal medium (33.7 mM Na_2_HPO_4_, 22.0 mM KH_2_PO_4_, 8.55 mM NaCl, 9.35 mM NH_4_Cl, 1 mM MgSO_4_, 0.3 mM CaCl_2_, trace elements solution (13.4 mM EDTA, 3.1 mM FeCl_3_-6H_2_O, 0.62 mM ZnCl_2_, 76 µM CuCl_2_-2H_2_O, 42 µM CoCl_2_-2H_2_O, 162 µM H_3_BO_3_, 8.1 µM MnCl_2_-4H_2_O), 1 µg/L biotin and 1 µg/L thiamin), and supplemented with propionic acid (1.50 g/L) and acetic acid (1.00 g/L) as main carbon sources (pH = 7.2).

### Strains isolation from biofilm sample

A total of 10 mL biofilm sample (three biological replicates) was placed into a 50 mL centrifuge tube containing 15 sterile glass beads (diameter 2 mm) and an equal volume of sterile phosphate-buffered saline solution (PBS). The sample was then homogenized using a vortex mixer for 30 s at 2500 rpm and serially diluted to 1 × 10^− 7^ with PBS. A 100 µL aliquot of each serial dilution was spread onto LB agar plates in triplicate because the standard wastewater medium is not available. LB is a common medium that can provide the nutrient to a majority of representative strains for cell growth. Certainly, some strains may prefer to grow in the wastewater and not with the LB medium, which were not considered in this study. Agar plates were incubated at 30 °C for two to five days. Colonies were selected by differentiable morphologies.

### Identification of isolated strains

Obtained strains were grown in fresh LB medium overnight and spread on LB plates three times to ensure pure cultures. Two-milliliter suspension of each pure culture was used for DNA extraction. DNA was extracted by using Genejet Genomic DNA Purification Kit according to the manufacturer’s instructions. Following DNA was used as a template to amplify and sequence the full 16S rRNA gene with primers (8F 5’ - AGAGTTTGATCCTGGCTCAG − 3’; 1492R 5’ - ACGGTTACCTTGTTACGACTT − 3’) by the Sanger method. Full sequences were checked and jointed manually using 4Peaks software (https://nucleobytes.com/4peaks/index.html). Resulting 16S rRNA gene sequences were queried and identified with reference sequences in the Silva database.

### Constructing the phylogenetic trees of isolated strains

For constructing the phylogenetic tree of these isolates, obtained 16S rRNA gene sequences were aligned and processed by MEGAX software (Kumar et al. 2018). The evolutionary relationship was inferred by using the Maximum Likelihood method and the Tamura-Nei model. To uncover the phylogenetic evolution relationship of single strains within the genera, reference sequences were downloaded from the NCBI database and aligned with the resulting sequences. The phylogenetic trees of single strains were constructed as described above.

### Screening multi-species biofilm formation by using crystal violet (CV) assay

Ten representative strains from four phyla were selected for the co-existence assay, which was performed with ten successive cycles of repeated batch cultivation. The purpose of this assay was to further screen multi-species with potential synergy to form biofilm. Obtained four strains grown as single- and multi-species by using a Nunc-TSP lid culture system, which comprises a 96-well plate lid with pegs extending into each well. Pre-cultures were grown up to OD_600 nm_ 1.0 at 30 °C. The cell suspensions were then adjusted to 10^8^ cells per milliliter in the synthetic medium. A total of 160 µL monoculture or mixed cultures (equal volume for each) were added to each well. Fresh medium was used as the negative control. The plates were sealed with Parafilm and incubated with shaking 200 rpm at 30 °C. Biofilm formation was quantified by crystal violet (CV) staining after 24 and 48 h of cultivation, as previously reported CV assays (Ren et al. 2014). CV quantification was performed on the pegs of the Nunc-TSP lid culture system. The peg lids were taken out and washed three times using PBS. Subsequently, the peg lids were placed in plates with 180 µL of an aqueous 1% (w/v) CV solution. The lids were rewashed three times with PBS after staining for 20 min. Peg lids were put into a new microtiter plate with 200 µL of 33% glacial acetic acid. After 15 min, the dissolved CV solution’s absorbance was determined at 590 nm using a microplate reader (Tecan, Spark).

### Inhibition assay of cell proliferation

The cell suspensions were adjusted to 10^8^ cells per milliliter in the synthetic medium. A total of 200 µL monoculture or mixed cultures (equal volume for each) were added to each well of 96-well plates. Different final concentrations of metabolic inhibitors (ST, 3BP, and 3-NP) were supplied to the bacterial suspension from 2 µg/mL to 256 µg/mL. No treatment was used as a control. Then the plate with cultures was placed on the shaker using 200 rpm at 30 °C. Cell proliferation was measured based on the net increase of the OD_600 nm_ value using a microplate reader (Tecan, Spark) after 24 and 48 h.

### Inhibition assay of biofilm formation

A total of 160 µL bacterial suspension (1 × 10^8^ cells/mL) in terms of monoculture and mixed cultures were prepared as above, then added to the Nunc-TSP lid culture system. Following metabolic inhibitors and their combinations were supplied to the suspension with different concentrations from 2  to 256 µg/mL, and no inhibitor addition was as the control. The plates were then placed on the shaker at 200 rpm and 30 °C. The biofilm quantification assays were performed as described above. The CV absorbance at 590 nm was used to evaluate the inhibiting effect on biofilm formation.

### Statistical analysis

All experiments were performed at least with four biological replicates in this study. One-way ANOVA followed by post hoc Tukey’s HSD tests were used for establishing the statistical differences of biofilm-forming capabilities. The significance level was set to *p* < 0.05.

## Results

### Isolation and identification of strains from biofilm

**Fig. 1:**
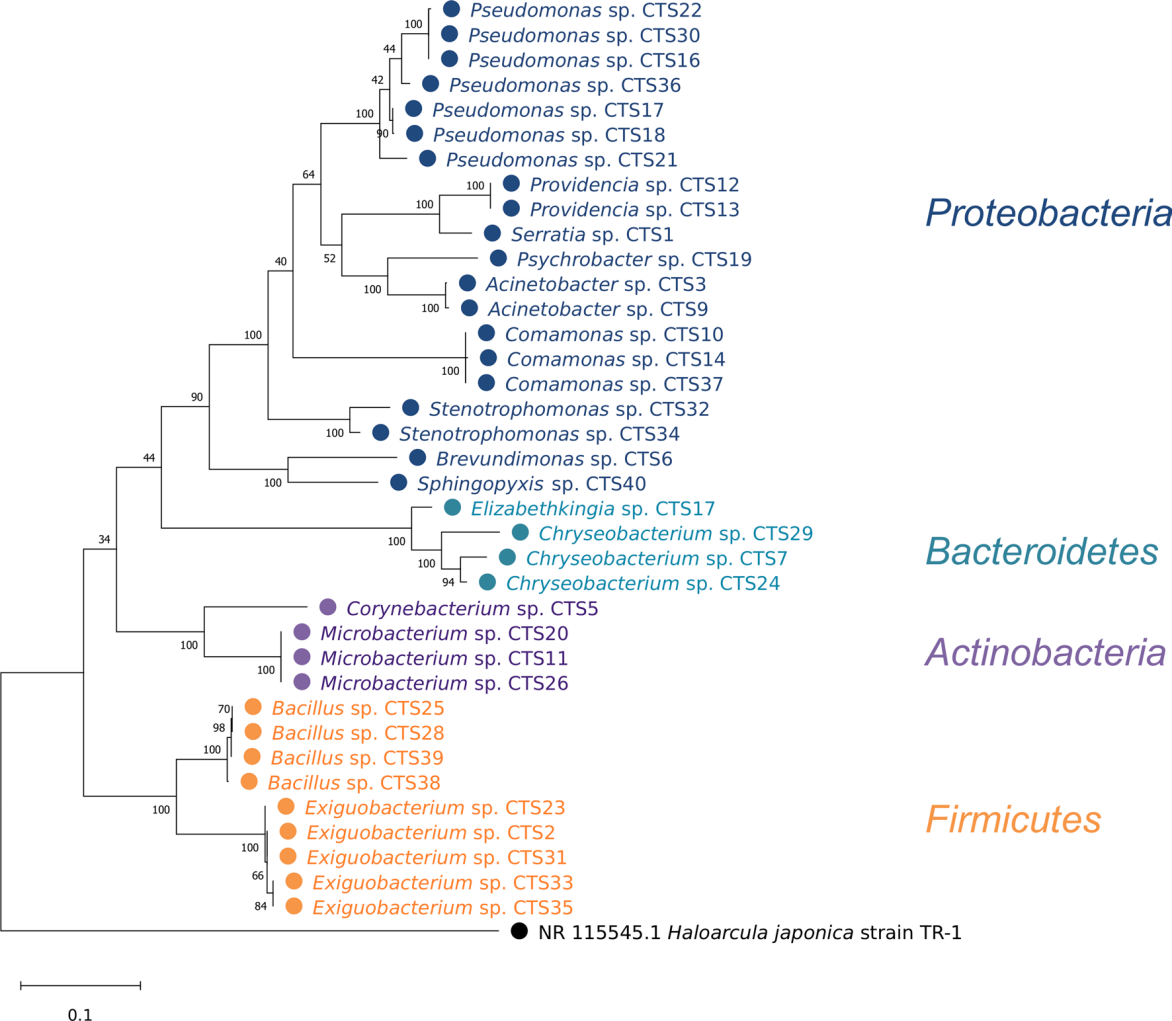
Phylogenetic tree of the strains isolated from biofilm sample. *Halocarcula japonica* strain TR-1 (GenBank: NR115545.1) was set as the outgroup species. Bootstrap values are displayed at each node

The viable strains that displayed distinct morphological features were isolated and identified from the cooling tower biofilm sample. Thirty-seven strains were found to belong to four phyla: *Actinobacteria*, *Proteobacteria*, *Bacteroidetes*, and *Firmicutes* (Fig. 1). Twenty strains were identified as *Proteobacteria*, consisting of nine genera, of which *Pseudomonas* was the most extensive branch. To further obtain a simplified microbial community with the capacity to use volatile fatty acids collectively, ten representative strains from the four phyla were selected to co-culture in a continuous mode. A total of four species, including *Acinetobacter* sp. CTS3 (A3), *Corynebacterium* sp. CTS5 (C5), *Providencia* sp. CTS12 (P12), and *Pseudomonas* sp. CTS17 (P17) were verified to be a stable coexistence over time. Phylogenic trees of each strain were constructed to explore the evolutionary relationship within the genus (Additional file 1: Fig. S1–S4). The result shows that the closest evolutionary relationship of A3 is *Acinetobacter johnsonii* (Additional file 1: Fig. S1). Similarly, *Pseudomonas composti* was found to be the closest related species with P17 (Additional file 1: Fig. S4). For C5 and P12, the closest references are *Corynebacterium glutamicum* and *Providencia heimbachae*, respectively (Additional file 1: Figs. S2 and S3).

### Obtaining of multi-species biofilms with synergistic interaction

**Fig. 2:**
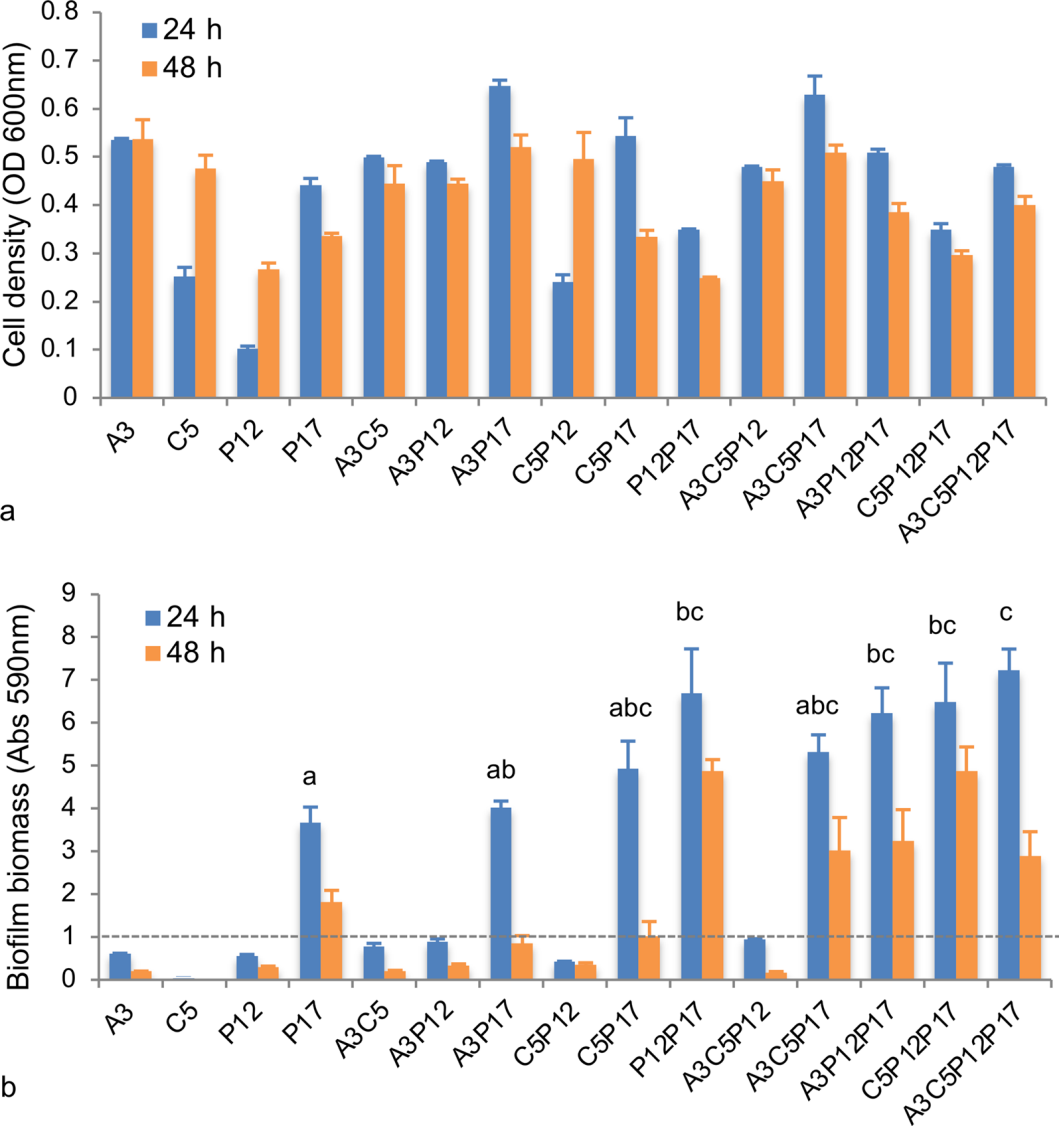
Comparison of cell density and biofilm biomass from mono- and multi-species cultures after 24 and 48 h.** a** Measurement of cell density. **b** Measurement of biofilm biomass. A3: *Acinetobacter* sp. CTS3, C5: *Corynebacterium* sp. CTS5, P12: *Providencia* sp. CTS12, and P17: *Pseudomonas* sp. CTS17. Lowercases mean significant differences of biofilm-forming capacity (above 1.0 (CV value)) among the cultures after 24 h, showing by different letters based on one-way ANOVA using post-hoc Tukey’s HSD test (*p* < 0.05). The detailed *p* values could be found in the Table S1

To obtain the multi-species biofilms and characterize their possible synergetic effect, all potential combinations of the four strains (single, dual-, triple-, and quadruple species) were cultured in the synthetic medium (Fig. 2a, b). Suspended cell density and biofilm-forming capacity were measured after 24 and 48 h. Four strains were found with different cell densities (OD_600 nm_) after the cultivation, suggesting that they can grow independently but exhibit different capacities to assimilate the volatile fatty acids. For all mixture cultivations, the A3P17 pair was presented with the highest cell density (OD_600 nm_: 0.65) after 24 h. The other mixtures’ cell density range was between 0.24 and 0.63 after 24 h. A3, C5, and P12 cultures showed a low capacity to form biofilm, with CV values (Abs_590 nm_) below 0.25. In contrast, the CV value from P17 culture showed a higher than 10-fold increase displaying a strong capacity for biofilm production. It is worth noting that all of the bacterial combinations comprising P17 possessed a strong ability to form a biofilm. The latter was predominantly observed in the four-species community. The CV value reached up to 8.30 after 24 h, suggesting an evident synergistic effect on biofilm formation among these species.

### Inhibitory effect of ST, 3BP, and 3-NP on cell proliferation

**Fig. 3:**
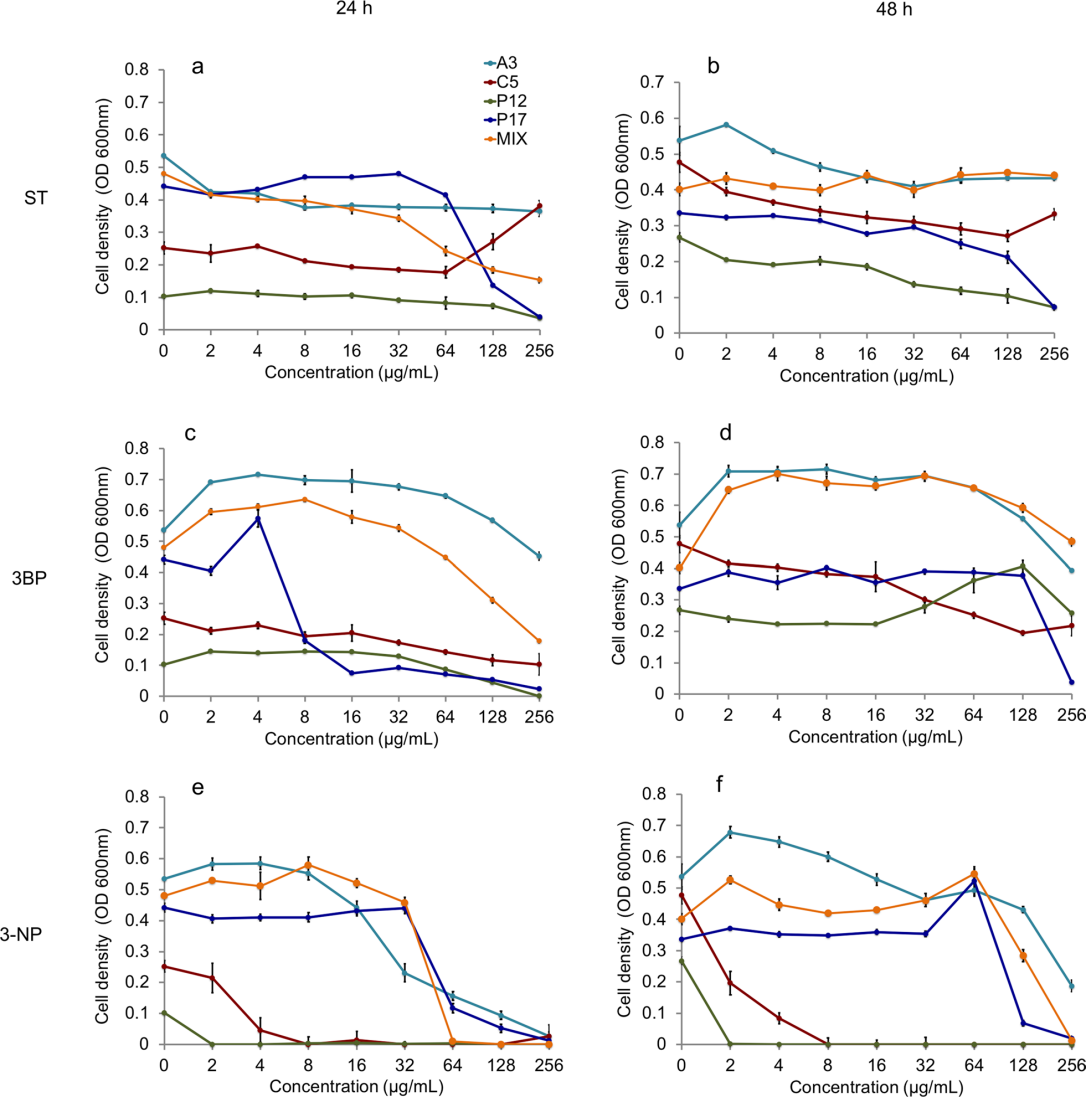
Cell density of monoculture (A3, C5, P12, and P17) and four species (MIX) culture with different metabolic inhibitors after 24 and 48 h.** a** Measurement of cell density with gradient concentrations of ST (sulfathiazole) (24 h). **b** Measurement of cell density with gradient concentrations of ST (48 h). **c** Measurement of cell density with gradient concentrations of 3BP (3-bromopyruvic acid) (24 h). **d** Measurement of cell density with gradient concentrations of 3BP (48 h). **e** Measurement of cell density with gradient concentrations of 3-NP (3-nitropropionic acid) (24 h). **f** Measurement of cell density with gradient concentrations of 3-NP (48 h). n = 4

Three chemical agents, i.e., sulfathiazole (ST), 3-bromopyruvic acid (3BP), and 3-nitropropionic acid (3-NP), were applied as the metabolic inhibitors to act on the cell proliferation of mono- and multi-species (four species). Cell density after 24 and 48 h cultivation was recorded with the treatment of different concentrations (Fig. 3). Three agents displayed different inhibitory effects on cell proliferation among four mono-species. ST cannot inhibit any of them entirely below the concentration of 256 µg/mL, but it showed a visible inhibiting effect to P17, especially as the concentration above 64 µg/mL (Fig. 3a, b). 3BP was able to prevent the cell proliferation of P12 and P17 almost entirely after 24 h with a concentration of 256 µg/mL, following the inhibiting effect was still acting on P17, but not P12 after 48 h (Fig. 3c, d). Notably, 3-NP presented a more substantial inhibitory effect than the other two (ST, 3BP) on these species (Fig. 3e, f). The cell proliferation of C5 and P12 was inhibited completely with concentrations of 2 µg/mL and 8 µg/mL, respectively. Additionally, cell proliferation of multi-species showed a distinct performance with three metabolic inhibitors. Cell density was reduced along with increasing concentrations of ST and 3BP after 24 h, while it was not affected even higher than the control after 48 h. Nevertheless, 3-NP still displayed an effective inhibition to the multi-species in 24 h (64 µg/mL) and 48 h (256 µg/mL), which was able to inhibit them fully.

### Inhibitory effect of ST, 3BP, and 3-NP on biofilm formation

**Fig. 4:**
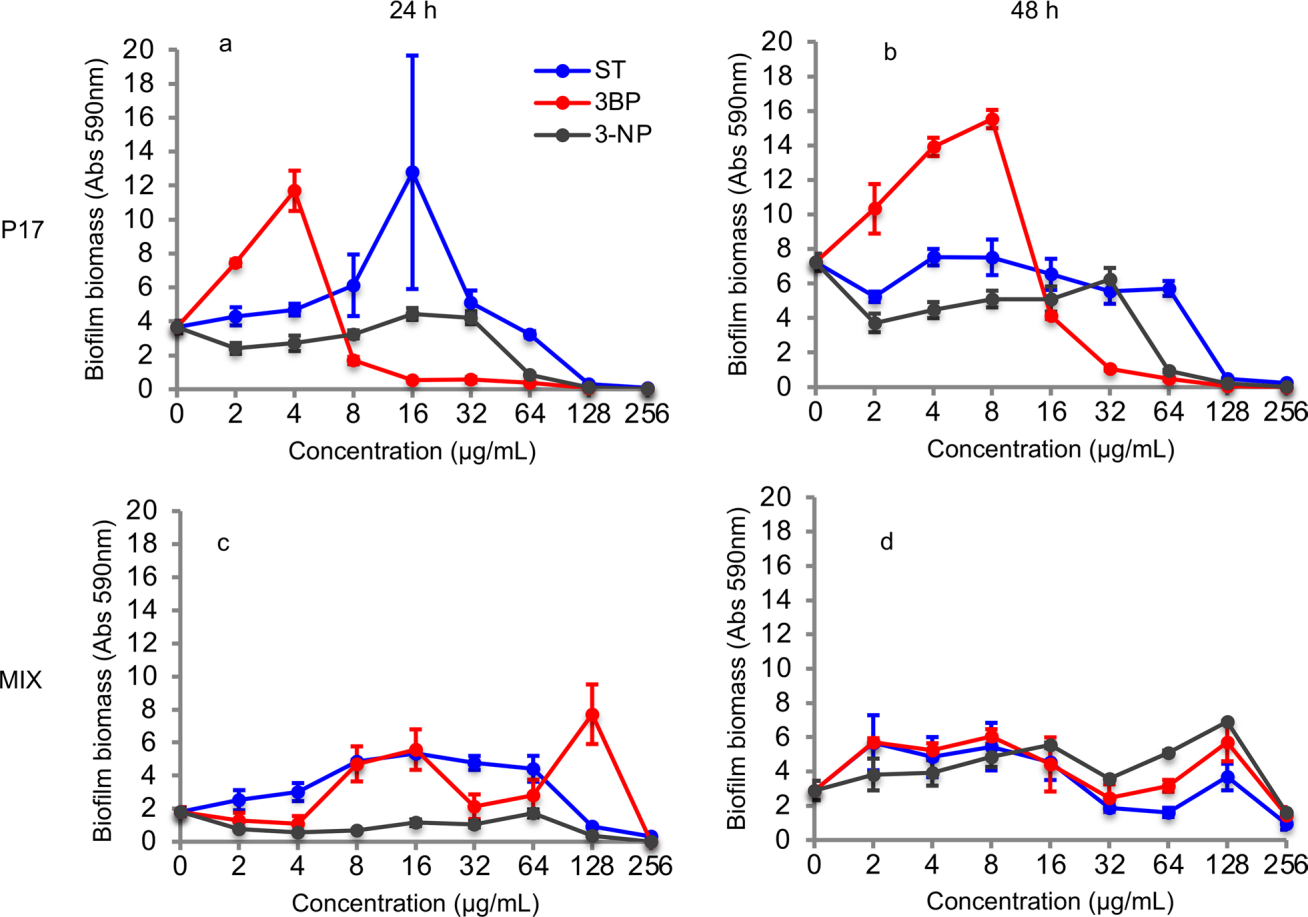
Biofilm formation of P17 and four species (MIX) cultures with different metabolic inhibitors after 24 and 48 h.** a** Biofilm formation of P17 culture with different metabolic inhibitors (24 h). **b** Biofilm formation of P17 culture with different metabolic inhibitors (48 h). **c** Biofilm formation of four species culture with different metabolic inhibitors (24 h). **d** Biofilm formation of four species culture with different metabolic inhibitors (48 h). ST: sulfathiazole, 3BP: 3-bromopyruvic acid, and 3-NP: 3-nitropropionic acid, n = 4

Serial concentrations of ST, 3BP, and 3-NP were added to P17 and multi-species (four species) cultures to evaluate their inhibitory effect on biofilm formation (Fig. 4). Three inhibitors displayed different inhibition patterns on the biofilm forming capacity of P17. This inhibitory effect was proved to increase along with increasing inhibitor concentrations, from 64 µg/mL up to 256 µg/mL. On the other hand, low inhibitor concentrations seem to boost biofilm formation, especially with 3BP. Notably, more than 95% reduction on biofilm formation was achieved with 3BP after 24 h at a concentration of 16 µg/mL and after 48 h at a concentration of 32 µg/mL. 3-NP and ST also were able to inhibit the biofilm formation effectively within concentrations of 64 µg/mL and 128 µg/mL. Moreover, these inhibitors displayed an inhibitory effect on multi-species growth. 3-NP showed the highest inhibitory capacity even at low concentrations of 2 µg/mL after 24 h, while biofilm was formed after 48 h. Similarly, multi-species biofilms were enhanced with other two inhibitors after 48 h compared to the biofilm quantification at 24 h. Despite the latter, biofilm formation was almost inhibited after 24 and 48 h when the inhibitor concentration reached 256 µg/mL.

### Enhancement of inhibitory effect on biofilm formation via combined inhibitors

**Fig. 5:**
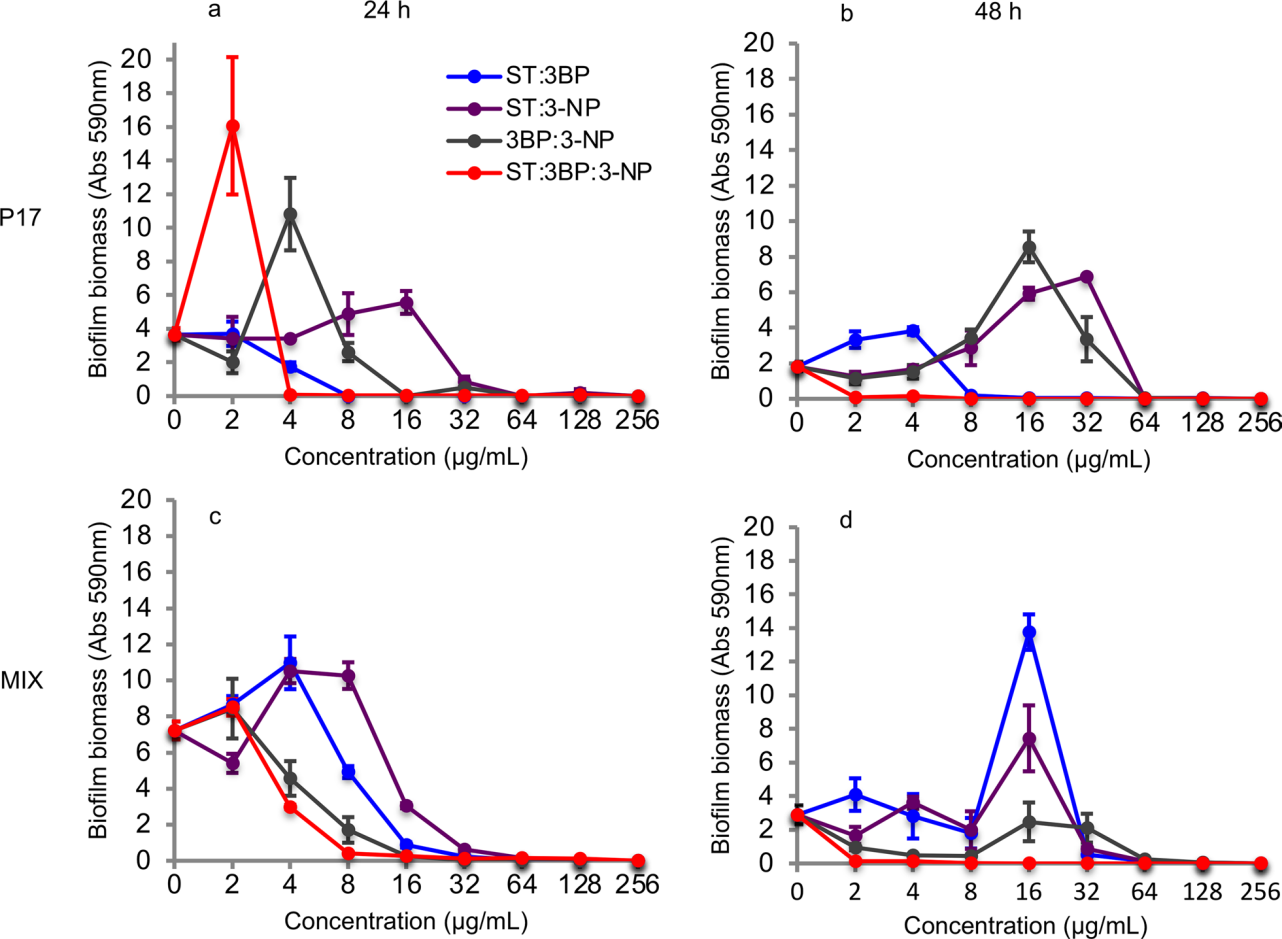
Biofilm formation of P17 and four species (MIX) cultures with combined metabolic inhibitors after 24 and 48 h.** a** Biofilm formation of P17 culture with combined metabolic inhibitors (24 h). **b** Biofilm formation of P17 culture with combined metabolic inhibitors (48 h). **c** Biofilm formation of four species culture with combined metabolic inhibitors (24 h). **d** Biofilm formation of four species culture with combined metabolic inhibitors (48 h). ST: sulfathiazole, 3BP: 3-bromopyruvic acid, and 3-NP: 3-nitropropionic acid, n = 4

Additionally, combinations of ST, 3BP, and 3-NP were used against the mono- and multi-species to investigate the enhancement effect. Inhibitor combinations showed a remarkable inhibitory effect on biofilm formation in P17 and multi-species (four species) cultures (Fig. 5). Dual inhibitors combination (ST and 3BP) inhibited P17 biofilm formation almost wholly at a concentration of 8 µg/mL within 48 h. These results show a remarkably higher inhibitory effect from simultaneous utilization of ST and 3BP compared to their independent usage (ST: 128 µg/mL, 3BP: 64 µg/mL). Similarly, the other dual-inhibitor combinations (ST:3-NP and 3BP:3-NP) also presented strong inhibitory effects on biofilm formation. They were able to inhibit the biofilm-forming of P17 and multi-species entirely with a concentration of 64 µg/mL after 24 and 48 h, of which the concentration was found to be lower than their individual usage. Notably, the combination of three inhibitors displayed an extreme inhibitory efficiency, which stopped the biofilm-forming of P17 and multi-species completely by treating with concentrations as low as 4 µg/mL and 8 µg/mL, respectively. Only low biofilm formation was detected even after 96 h; it showed that the combined inhibitors were able to impact the biofilm-forming persistently (Additional file 1: Figure S5). These results demonstrate that an enhancement effect exists among the inhibitors, especially for combining three of them. The latter and their inhibition characterization make it clear that they could be part of a promising regulated strategy to prevent biofilm formation of mono- and multi-species.

## Discussion

In this study, cultivable bacterial isolates were identified from a biofilm sample originating from a cooling tower. The capacity of biofilm formation among representative species was evaluated, showing the synergistic effect of biofilm formation among four species. Three metabolic inhibitors (ST, 3BP, and 3-NP) were selected against cell proliferation and biofilm formation owing to their ability to target vital metabolic pathways in cellular metabolism. They displayed distinct inhibitory effects on cell proliferation, and all of them could inhibit the biofilm development with concentrations lower than 256 µg/mL. Notably, combining three inhibitors possessed potent inhibition with a much low concentration (8 µg/mL) against the mono- and multi-species biofilms for a long time. Targeting cellular metabolism with distinct functional metabolic inhibitors turns into a promising approach to prevent multi-species biofilm formation.

Many studies have reported the microbial composition from cooling water systems. A combined NGS-based approach was used to study the whole bacterial community from a cooling tower, which displayed a highly diverse bacterial community with more than 808 genera observed (Pereira et al. 2017). Seven species producing extracellular polymeric substances were isolated and characterized from the cooling tower through sequential culturing in an enriched medium (Ceyhan and Ozdemir 2008). 37 bacterial strains were identified in the cooling water biofilm mainly belonging to *Proteobacteria* and *Firmicutes*, which occupied more than 80% of the total isolates in this study. *Proteobacteria* has been identified as the predominant bacteria in biofilms growing on recirculating cooling-water systems (MacDonald and Brözel 2000; Pinel et al. 2020). *Firmicutes* and *Proteobacteria* were described as the most dominant phyla in the ecosystems of cooling towers within sugar-cane processing plants (Sharmin et al. 2013). In fact, most of the identified strains such as *Pseudomonas* sp., *Acinetobacter* sp., *Corynebacterium* sp. have been detected from various wastewater environments (Greay et al. 2019; Han et al. 2020). Besides, *Providencia* sp. was isolated from wastewater and applied to perform microbial remediation (Abo-Amer et al. 2013). A frequent species, *Legionella*, was not detected, while *Legionella* was frequently observed in biofilms obtained from cooling water suspensions (Edagawa et al. 2008; Pereira et al. 2017). A previous study uncovered that *Pseudomonas* species had a robust negative correlation with *Legionella* in cooling towers (Paranjape et al. 2020), which may explain the lack of *Legionella* in the present microbial community. Overall, these isolates are likely to have social interaction related to biofilm formation, as they are living in such an environment.

Building a multi-species biofilm model has more ecological relevance compared to mono-culture model, which serves as an effective way to explore biofilm development in natural conditions (Røder et al. 2016). A high prevalence of synergistic effects was observed from different isolates during biofilm development (Liu et al. 2019; Ren et al. 2015). In the present work, four species were able to assimilate and tolerate the supplied volatile fatty acids in the synthetic medium. In fact, volatile fatty acids can also inhibit cell growth or display distinctive toxic effects (Pinhal et al. 2019; Wilbanks and Trinh 2017). Except for their growth capabilities, P17 was found to form biofilm when cultured alone. *Pseudomonas* species are frequent inhabitants in freshwater environments via adhesion and biofilm formation (Pereira et al. 2018). The result confirms the vital role of *Pseudomonas* species during the biofilm forming process in the cooling tower. Intriguingly, biofilm biomass of the dual-/ triple- and four species was promoted when P17 was present, showing a synergistic effect between these biofilm production strains. Previous studies have demonstrated that *Pseudomonas* had diverse molecular interactions with *Acinetobacter* sp. (Hansen et al. 2007) and *Corynebacterium* sp. (Brathwaite and Dickey 1970). Nevertheless, synergic mechanisms among the four species are still not clear, further exploration is needed to reveal the synergy for biofilm formation.

Recently, plentiful small-molecule agents have been developed to treat microbial biofilms based on their mechanistic understanding (Qvortrup et al. 2019). In this study, three metabolic inhibitors were selected as they can target distinct metabolic pathways. ST was found with the broadest activity spectrum against microbial biofilms, which has been selected to prevent multi-species biofilm formation (Parijs and Steenackers 2018). It has been reported to interfere with c-di-GMP biosynthesis, thereby reducing biofilm formation effectively (Antoniani et al. 2010). c-di-GMP is used as a signaling system with a crucial role in regulating bacterial behavior and especially for biofilm formation (Jenal et al. 2017). Therefore, the potential mechanism that ST prevents biofilm formation could be inhibiting c-di-GMP biosynthesis (Fig. 4). 3BP and 3-NP also displayed the visible inhibiting effect to the biofilm formation (Fig. 4). Previous studies showed that 3BP and 3-NP were the enzyme inhibitors to succinate dehydrogenase and isocitrate lyase, which are essential enzymes from metabolic pathways of volatile fatty acids catabolism (Moynihan and Murkin 2014; Sharma et al. 2000). Furthermore, 3BP has been described as a potent inhibitor to glycolysis/gluconeogenesis (Darabedian et al. 2018), which is related to converting the intermediates from volatile fatty acids catabolism to biofilm precursors (monosaccharides, eDNA). These could be the reasons that 3BP and 3-NP are able to prevent cell growth and biofilm formation. Particularly, the inhibitory efficiency to biofilm development was remarkably improved when using three metabolic inhibitors simultaneously, implying that they targeted different metabolic pathways related to biofilm forming.

Unexpectedly, the biofilm formation of P17 and multi-species were promoted under relatively low concentration of the metabolic inhibitors, for instance, treating P17 with ST (16 µg/mL) after 24 h (Fig. 4a), treating multi-species with ST:3BP (16 µg/mL) after 48 h (Fig. 5d). In fact, some antibiotic’s subinhibitory concentrations have been found to promote microbial biofilm formation by a trade-off between drug toxicity and the beneficial results of cell lysis (Yu et al. 2018). It suggests that the trade-off of inhibitor effect and beneficial factors is present when using metabolic inhibitors to prevent biofilm formation. Further inquiry into the interplay between bacterial response and inhibitor efficacy in biofilm development is necessary to determine each inhibitor’s optimal conditions. Our results indicated that combined the three metabolic inhibitors prevented the biofilm formation for more than 96 h (Figure S5). In fact, many metabolic inhibitors can be degraded by particular microorganisms; for instance, *Phanerochaete chrysosporium* is capable of removing ST (Kwak et al. 2017). Further investigation of their degradation rates and stability is necessary to ensure long-term efficacy.

Overall, we isolated the biofilm-forming related microorganisms and developed a multi-species biofilm model by mimicking a natural cooling water system. Culturing results showed that four species were able to assimilate the volatile fatty acids and generate biofilm biomass with a synergistic effect. Combined metabolic inhibitors displayed an effective inhibitory effect on biofilm development, which demonstrated that targeting cellular metabolism is a potent approach to inhibit synergistic biofilm formation of multi-species. Exploring the bacterial responses to these metabolic inhibitors and building more complex biofilm systems will help evaluate their inhibiting efficacy in practice.

## Supplementary Information

**Additional file 1.** Additional figures.

## Data Availability

Sequencing data are available at the NCBI GenBank database with the accession numbers MW389057 – MW389093, including *Acinetobacter* sp. CTS3 (MW389059), *Corynebacterium* sp. CTS5 (MW389060), *Providencia* sp. CTS12 (MW389066), and *Pseudomonas* sp. CTS17 (MW389070). In addition, the major bacterial strains have been deposited into the public BCCM/LMG Bacteria Collection with the accession numbers: *Acinetobacter* sp. CTS3 (LMG 32,410), *Corynebacterium* sp. CTS5 (LMG 32,411), *Providencia* sp. CTS12 (LMG 32,412), and *Pseudomonas* sp. CTS17 (LMG 32,413).

## Abbreviations

ST: Sulfathiazole
3BP: 3-bromopyruvic acid
3-NP: 3-nitropropionic acid
c-di**-**GMP: Cyclic di-GMP
LB: Lysogeny broth
CV: Crystal violet
A3: *Acinetobacter* sp. CTS3
C5: *Corynebacterium* sp. CTS5
P12: *Providencia* sp. CTS12
P17: *Pseudomonas* sp. CTS17
