# Supplementary material for: Combined utilization of metabolic inhibitors to prevent synergistic multi-species biofilm formation
Source: AMB Express. 2022 Mar 4;12:32. doi: 10.1186/s13568-022-01363-4 (PMC8897544; doi:10.1186/s13568-022-01363-4)
Supplement: Supplementary file 1 — Additional file 1. Additional figures. [file 13568_2022_1363_MOESM1_ESM.docx]

***Supplementary material***

Journal: AMB Express

**Combined utilization of metabolic inhibitors to prevent synergistic multi-species biofilm formation**

**Dingrong Kang^1*^, Wenzheng Liu^12^, Fatemeh Bajoul Kakahi^1^, Frank Delvigne^1*^**

^1^TERRA Research and Teaching Centre, Microbial Processes and Interactions (MiPI), Gembloux Agro-Bio Tech, University of Liège, Gembloux 5030, Belgium

^2^School of Food and Pharmaceutical engineering, Nanjing Normal University, Nanjing 210000, China

***Corresponding authors:**

Dingrong Kang: [dingrong.kang@hotmail.com](mailto:dingrong.kang@hotmail.com); Tel: +32 81622309; Fax: +32 81614222

Frank Delvigne: [f.delvigne@uliege.be](mailto:f.delvigne@uliege.be); Tel: +32 81622309; Fax: +32 81614222

**(Figures S1 – S5 and Table S1)**

**Figure S1. Phylogenetic tree of *Acinetobacter* sp. CTS3 (A3).** *Escherichia coli* (Genbank: J01859.1) was set as the outgroup species. Bootstrap values are displayed at each node.

**Figure S2. Phylogenetic tree of *Corynebacterium* sp. CTS5 (C5).** *Escherichia coli* (Genbank: J01859.1) was set as the outgroup species. Bootstrap values are displayed at each node.

**Figure S3. Phylogenetic tree of *Providencia* sp. CTS12 (P12).** *Escherichia coli* (Genbank: J01859.1) was set as the outgroup species. Bootstrap values are displayed at each node.

**Figure S4. Phylogenetic tree of *Pseudomonas* sp. CTS17 (P17).** *Actinomyces masslliensis* was set as the outgroup species. Bootstrap values are displayed at each node.

**
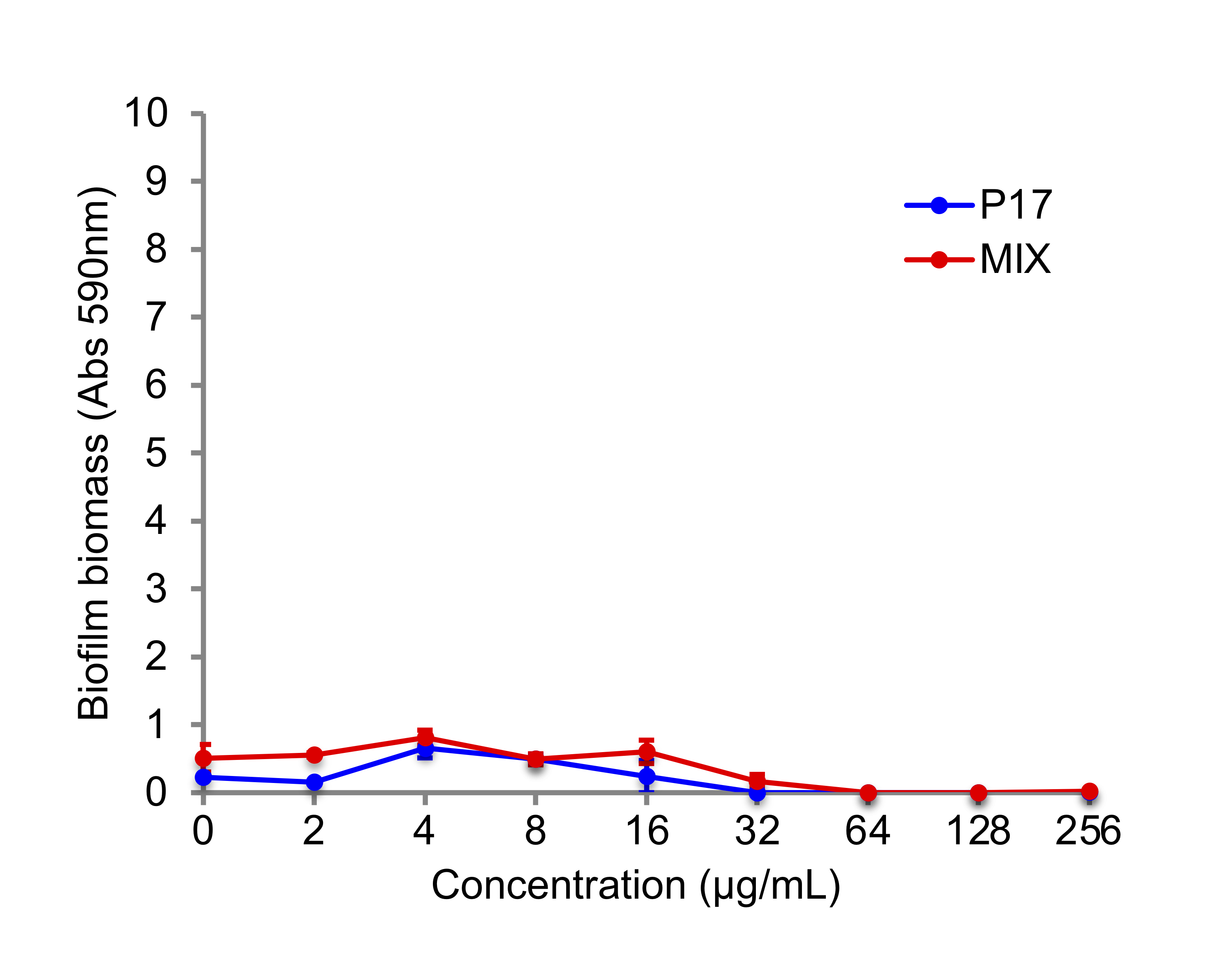
**

**Figure S5. Biofilm formation of P17 and four species (MIX) with the treatment of three metabolic inhibitors (ST: 3BP: 3-NP) in the synthetic medium after 96 h, n = 4.**

**Table S1. Comparison of biofilm-forming capacity among different cultures based on one-way ANOVA followed by post hoc Tukey’s HSD tests.**

| **Culture 1** | **Culture 2** | ***p* value (tukey )** |
| --- | --- | --- |
| **A3C5P12P17** | A3C5P17 | 0.279 |
|  | A3P12P17 | 0.914 |
|  | A3P17 | **0.005** |
|  | C5P12P17 | 0.982 |
|  | C5P17 | 0.106 |
|  | P12P17 | 0.998 |
|  | P17 | **< .001** |
| **A3C5P17** | A3P12P17 | 0.974 |
|  | A3P17 | 0.848 |
|  | C5P12P17 | 0.906 |
|  | C5P17 | 1 |
|  | P12P17 | 0.803 |
|  | P17 | 0.438 |
| **A3P12P17** | A3P17 | 0.264 |
|  | C5P12P17 | 1 |
|  | C5P17 | 0.848 |
|  | P12P17 | 1 |
|  | P17 | **0.042** |
| **A3P17** | C5P12P17 | 0.154 |
|  | C5P17 | 0.974 |
|  | P12P17 | **0.092** |
|  | P17 | 1 |
| **C5P12P17** | C5P17 | 0.694 |
|  | P12P17 | 1 |
|  | P17 | **0.018** |
| **C5P17** | P12P17 | 0.542 |
|  | P17 | 0.748 |
| **P12P17** | P17 | **0.009** |
